# Supplementary material for: Mustard Meal Extract as an Alternative to Zinc Oxide for Protecting the Intestinal Barrier Against E. coli-Lipopolysaccharide Damage
Source: Int J Mol Sci. 2024 Dec 31;26(1):273. doi: 10.3390/ijms26010273 (PMC11719691; doi:10.3390/ijms26010273)
Supplement: Supplementary file 1 [file ijms-26-00273-s001.zip › ijms-3352315-supplementary.pdf]

Supplementary Table S1. List and characteristics of target genes detected in qPCR array.

No crt	Species	Accession No	Gene abbreviation	Gene name	qPCR amplicon size (bp)
1	Homo sapiens	NM_003263	*TLR1*	toll like receptor 1	97
2	Homo sapiens	NM_001318790.1	*TLR2*	toll like receptor 2	152
3	Homo sapiens	NM_003265	*TLR3*	toll like receptor 3	354
4	Homo sapiens	NM_138554	*TLR4*	toll like receptor 4	210
5	Homo sapiens	NM_003268	*TLR5*	toll like receptor 5	148
6	Homo sapiens	NM_006068.4	*TLR6*	toll like receptor 6	152
7	Homo sapiens	NM_016562	*TLR7*	toll like receptor 7	113
8	Homo sapiens	NM_138636.4	*TLR8*	toll like receptor 8	156
9	Homo sapiens	NM_017442.3	*TLR9*	toll like receptor 9	78
10	Homo sapiens	NM_030956	*TLR10*	toll like receptor 10	152
11	Homo sapiens	NM_004620	*TRAF-6*	TNF receptor associated factor 6	161
12	Homo sapiens	NM_001569	*IRAK1*	interleukin 1 receptor associated kinase 1	158
13	Homo sapiens	NM_145331	*TAK*	mitogen-activated protein kinase kinase kinase 7 (MAP3K7)	107
14	Homo sapiens	NM_002737	*PKCA*	protein kinase C, alpha subunit	91
15	Homo sapiens	NM_006254	*PKCD*	protein kinase C, delta subunit	106
16	Homo sapiens	NM_001243984	*NF-kB/p65*	RELA proto-oncogene, NF-kB subunit	109
17	Homo sapiens	NM_002468	*MyD88*	Myeloid differentiation primary response protein 88	142
18	Homo sapiens	NM_015364	*MD-2*	myeloid differentiation factor 2	137
19	Homo sapiens	NM_019009	*Tollip*	toll interacting protein	139
20	Homo sapiens	NM_021101	*CLDN1*	claudin 1	129
21	Homo sapiens	NM_001171092	*CLDN2*	claudin 2	88
22	Homo sapiens	NM_001305.4	*CLDN4*	claudin 4	94

23	Homo sapiens	NM_001130861.1	*CLDN5*	claudin 5	112
24	Homo sapiens	NM_001146077	*CLDN14*	claudin 14	122
25	Homo sapiens	NM_001001346.3	*CLDN20*	claudin 20	132
26	Homo sapiens	NM_194284.2	*CLDN23*	claudin 23	101
27	Homo sapiens	NM_001205254	*OCCL*	occludin	132
28	Homo sapiens	NM_003257.3	*ZO-1*	Zonula Occludens-1	148
29	Homo sapiens	NM_016946.4	*JAM-A*	junctional adhesion molecule A (F11 receptor)	118
30	Homo sapiens	NM_012301.4	*MAGI2*	membrane associated guanylate kinase, WW and PDZ domain containing 2	142
31	Homo sapiens	NM_002070	*GNAI2*	G protein subunit alpha i2	140
32	Homo sapiens	NM_004145.4	*MIO9B*	myosin IXB	119
33	Homo sapiens	NM_182493	*MLCK*	myosin light chain kinase 3	156
34	Homo sapiens	NM_002828	*PTPN2*	protein tyrosine phosphatase non-receptor type 2	131
35	Homo sapiens	NM_002457.4	*MUC2*	mucin 2	135
36	Homo sapiens	NM_004425	*ECM*	extracellular matrix protein 1	148
37	Homo sapiens	NM_015456.5	*NELF*	negative elongation factor complex member B	123
38	Homo sapiens	NM_005406	*ROCK 1*	Rho associated coiled-coil containing protein kinase 1	157
39	Homo sapiens	NM_006092	*NOD1*	nucleotide binding oligomerization domain containing 1	135
40	Homo sapiens	NM_003821	*RIPK2*	receptor interacting serine/threonine kinase 2	133
